# Effects of nursing intervention on lung infection prevention in patients with tracheotomy

**DOI:** 10.1097/MD.0000000000017063

**Published:** 2019-09-13

**Authors:** Hua Li, Xiao-Hong Mao

**Affiliations:** a Department of Nursing Care; b Department of Infection Control, Hanzhong People's Hospital, Hanzhong, China.

**Keywords:** lung infection, nursing intervention, randomized controlled trial, tracheotomy

## Abstract

**Background:**

This study will evaluate the effects of nursing intervention (NIV) on lung infection prevention (LIP) in patients with tracheotomy.

**Methods:**

The electronic databases of MEDLINE, Cochrane Library, EMBASE, Web of Science, Chinese Biomedical Literature Database, and China National Knowledge Infrastructure will be retrieved from inception to the June 1, 2019 for randomized controlled trials investigating the effects of NIV on LIP in patients with tracheotomy without any language limitations. In addition, we will also search grey literature to avoid missing any potential studies. Two independent authors will perform study selection, data extraction, and risk of bias evaluation.

**Results:**

This study will investigate the effects of NIV on LIP in patients with tracheotomy. The primary outcome is incidence of lung infection. The secondary outcomes include pulmonary function, quality of life, and complications post-surgery.

**Conclusion:**

The results of this study will summarize recent evidence for the effects of NIV on LIP in patients with tracheotomy.

No ethic approval is needed in this study, because it will not need any individual data. The results of this study will be published at a peer-reviewed journal.

## 1 Introduction

Tracheotomy is one of the most important managements of maintaining airway patency to rescue and treat critically ill patients.^[1–3]^ It consists of percutaneous dilatational tracheostomy and open surgical tracheostomy.^[4–5]^ However, such management often causes lung infection prevention (LIP), death or permanent disability.^[6–13]^ It has been estimated that it affects about 500 patients in the United States.^[14]^ Thus, it is very important and very necessary to prevent these complications, especially for LIP.

Several clinical studies are reported to prevent lung infection effectively, such as nursing intervention (NIV).^[15–18]^ However, no study has evaluated the effects of NIV on LIP in patients with tracheotomy systematically. Therefore, this study systematically assesses the effects of NIV on LIP in patients with tracheotomy.

## 2 Methods

### 2.1 Eligibility criteria

#### 2.1.1 Study types

This study will only include randomized controlled trials (RCTs) on exploring the effects of NIV on LIP in patients with tracheotomy. All other studies will be excluded, including non-clinical studies, non-controlled studies, and non-RCTs.

#### 2.1.2 Participant types

All patients with tracheotomy will be included in this study regardless the race, gender, and age.

#### 2.1.3 Intervention types

Experimental group: all patients receive NIV intervention.

Control group: patients can receive any interventions, except NIV.

#### 2.1.4 Outcome types

The primary outcome is incidence of lung infection. The secondary outcomes include pulmonary function, as measured by Peak Expiratory Flow, and related tools; quality of life, as measured by 36-Item Short Form Survey and relevant scales; and complications post-surgery.

### 2.2 Search methods

We will search the following electronic databases from inception to the June 1, 2019 for RCTs investigating the effects of NIV on LIP in patients with tracheotomy without any language limitations: MEDLINE, Cochrane Library, EMBASE, Web of Science, Chinese Biomedical Literature Database, and China National Knowledge Infrastructure. The search strategy for MEDLINE is exerted in Table 1. Similar strategies will also be applied to any other electronic databases. In addition, we will also search conference proceedings, clinical trials registry, and reference lists of associated reviews.

**Table 1:**
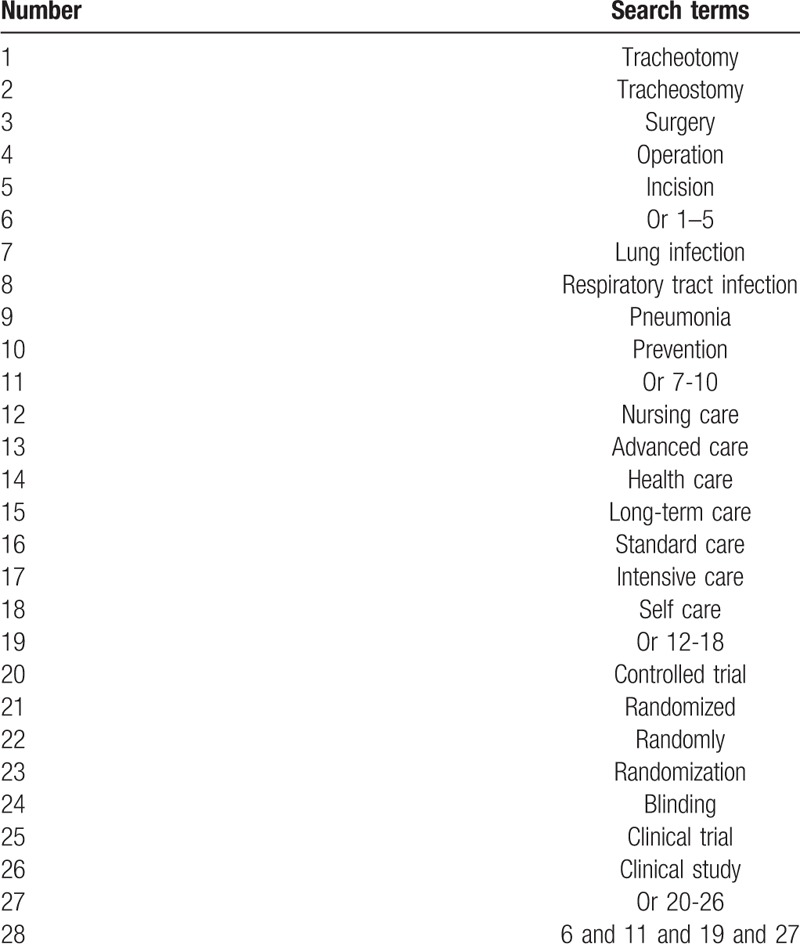
Specific search strategy for MEDLINE database.

### 2.3 Data collection

#### 2.3.1 Study selection

Two authors will independently select studies according to the predefined eligibility criteria. Any disagreements will be solved by a third author through discussion. NoteExpress 3.2.0 software will be used to manage the literature records. Two steps will be included in the process of study selection. First, all the records will be read by the titles and abstracts, and irrelevant studies will be excluded. Second, full texts will be read to further assess if they meet all the eligibility criteria. The reason for each excluded study will be noted. The process of study selection is presented in a flowchart of Preferred Reporting Items for Systematic Reviews and Meta-Analyses.

#### 2.3.2 Data extraction

Two authors will independently carry out data extraction in accordance with the predefined data extraction sheet. Any divergences will be resolved by a third author via discussion. The extracted information includes characteristics of study and patients, such as title, authors, year of publication, location, gender, etc; study methods, such as randomization, concealment, blinding, etc; treatment details, such as dosage, frequency, duration, etc; and outcome measurements, such as primary, secondary outcomes, safety, etc.

#### 2.3.3 Missing data management

If there is any missing or insufficient information, we will contact primary authors using email. If we cannot receive this information, we will analyze the current available data only, and will discuss its potential affects.

### 2.4 Risk of bias evaluation

Two authors will independently evaluate the risk of bias for all included studies using Cochrane Risk of Bias Tool. A third author will be invited to solve any divisions regarding risk of bias evaluation between two authors. It comprises of 7 fields, and each one has 3 items of low, unclear, and high risk of bias.

### 2.5 Data synthesis and analysis

ReMan 5.3 software will be used to carry out statistical analysis. As for continuous data, they will be expressed as mean difference or standardized mean difference with 95% confidence intervals. As for dichotomous data, they will be expressed as risk ratio with 95% confidence intervals.

We will utilize *I*^*2*^ test to check heterogeneity, which is interpreted as below: *I*^*2*^ ≤ 50 means low level of heterogeneity; *I*^2^ > 50% means high level of heterogeneity. If there is low heterogeneity, a fixed-effects model will be used, and meta-analysis will be carried out. If there is high heterogeneity, a random-effects model will be applied, and subgroup analysis will be conducted. If there is still high heterogeneity after subgroup analysis, data will not be pooled, and meta-analysis will not be carried out. Instead, narrative summary will be reported.

### 2.6 Additional analysis

Subgroup analysis will be carried out in accordance with the different characteristics, study quality, treatments, and outcome measurements. In addition, we will also carry out sensitivity analysis to check the robustness of pooled outcome results by removing high risk of bias studies.

### 2.7 Reporting bias

Funnel plot^[19]^ and Egger regression text^[20]^ will be used to identify any reporting bias if more than 10 studies are included.

## 3 Discussion

Although several clinical studies have reported that NIV can help to manage LIP in patients with tracheotomy, no study has evaluated its effects and safety on LIP for patients with tracheotomy. Thus, this study will assess the published RCTs evidence for the effects and safety of NIV for LIP in patients with tracheotomy. The findings of this study will summarize the latest evidence of NIV on LIP, and will inform our understanding of NIV on LIP in patients with tracheotomy.

## Author contributions

**Conceptualization:** Hua Li, Xiao-hong Mao.

**Data curation:** Hua Li, Xiao-hong Mao.

**Formal analysis:** Hua Li.

**Funding acquisition:** Xiao-hong Mao.

**Investigation:** Hua Li, Xiao-hong Mao.

**Methodology:** Hua Li.

**Project administration:** Xiao-hong Mao.

**Resources:** Hua Li.

**Software:** Hua Li.

**Supervision:** Xiao-hong Mao.

**Validation:** Hua Li, Xiao-hong Mao.

**Visualization:** Hua Li, Xiao-hong Mao.

**Writing – original draft:** Hua Li, Xiao-hong Mao.

**Writing – review & editing:** Hua Li, Xiao-hong Mao.
